# Sex differences in vaccine-induced humoral immunity

**DOI:** 10.1007/s00281-018-0726-5

**Published:** 2018-12-13

**Authors:** Stephanie Fischinger, Carolyn M. Boudreau, Audrey L. Butler, Hendrik Streeck, Galit Alter

**Affiliations:** 1 Ragon Institute of MGH, MIT, and Harvard, 400 Technology Square, Cambridge, MA 02139 USA; 2 Institut für HIV Forschung, Universität Duisburg-Essen, Duisburg, Germany

**Keywords:** Sex differences, Gender differences, Immune response, Vaccination, Hormones, Infection

## Abstract

Vaccines are among the most impactful public health interventions, preventing millions of new infections and deaths annually worldwide. However, emerging data suggest that vaccines may not protect all populations equally. Specifically, studies analyzing variation in vaccine-induced immunity have pointed to the critical impact of genetics, the environment, nutrition, the microbiome, and sex in influencing vaccine responsiveness. The significant contribution of sex to modulating vaccine-induced immunity has gained attention over the last years. Specifically, females typically develop higher antibody responses and experience more adverse events following vaccination than males. This enhanced immune reactogenicity among females is thought to render females more resistant to infectious diseases, but conversely also contribute to higher incidence of autoimmunity among women. Dissection of mechanisms which underlie sex differences in vaccine-induced immunity has implicated hormonal, genetic, and microbiota differences across males and females. This review will highlight the importance of sex-dependent differences in vaccine-induced immunity and specifically will address the role of sex as a modulator of humoral immunity, key to long-term pathogen-specific protection.

## Introduction

Vaccines are among the most impactful public health interventions, preventing millions of new infections and deaths annually worldwide [1]. Protection following vaccination depends on a coordinated response by multiple immune arms, collectively giving rise to durable immunity. While innate immunity and CD4 helper T cell profiles are linked to the generation of long-lived protective immunity and pathogen eradication, antibodies represent the primary correlate of protection following most clinically approved vaccines [2]. Antibodies can either directly neutralize pathogens or aid in destruction of opsonized pathogens via phagocytosis, activation of complement, or the recruitment of natural killer (NK) cells [3]. These non-neutralizing functions are induced via the binding of the antibody Fc-domain to Fc-receptors on innate immune cells, which induce immune functions. The importance of these non-neutralizing antibody-dependent effector functions has been corroborated across diseases. For example, the importance of Fc-effector functions was identified as a correlate of protection against HIV in the first protective RV144 vaccine trial, linked to antibody-dependent cellular cytotoxicity (ADCC) in the absence of neutralization [4]. Additionally, vaccination against malaria, Bordetella pertussis, and influenza have shown protection associated with antibody-dependent effector functions [5–8]. Along the same lines, while neutralization fails to predict influenza-vaccine efficacy [9], phagocytosis, activation of complement system, and cytotoxicity have all been associated with protection from lethal influenza challenge in mice, pointing to a broad array of antibody functions in protection from disease [5, 8, 10, 11]. Thus, while antibodies represent the primary correlate of protection, their mechanism of action may vary tremendously across disease and pathogen.

Despite our growing mechanistic appreciation for the role of antibodies in protection from infection/disease, emerging data point to variable vaccine responsiveness across populations, both with respect to magnitude and quality [12]. Specifically, genetics, race, age, social background, and sex have all been shown to influence the immune response against a given vaccine [13]. For example, particular human leukocyte antigens (HLA), involved in antigen presentation to T cells, have been linked to non-responsiveness to hepatitis B vaccination (HBV) [14], attributable to compromised T cell help for the induction of B cell responses. Additionally, polymorphisms and epigenetic changes in Toll-like receptor pathways, critical for innate sensing and arming the immune system, have also been shown to impact vaccine profiles [15]. Moreover, reduced magnitude of vaccine immunity has been observed with proximity to the equator, hypothesized to be linked to co-endemic disease burden and health status resulting in dampened responses to vaccination [16]. However, strikingly, even within the same genetic pool and environment, significant differences are consistently observed among the sexes following vaccination.

The term sex is defined as a person’s biological characteristics, such as sex chromosomes, hormone concentrations, and sex organ physiology. Conversely, gender describes cultural and social qualities that define a person as a man or woman [17]. The combination of social, cultural, and biological elements factoring into gender differences makes it difficult to differentiate between biological mechanisms and sex-specific behavior [18]. Accumulating data have shown that sex, rather than gender, is a critical predictor of susceptibility to particular infections and autoimmune diseases, but also strongly influences response to immunization [19]. In general, women are more resistant to bacterial and viral infections, linked to overall higher antibody levels as well as greater T cell activation [12]. However, as a consequence of this enhanced immune activation, women tend to experience more adverse reactions following vaccination and have a higher incidence of autoimmune disease [20]. Conversely, men are more susceptible to infectious diseases due to the hormone-dependent expression of cell receptors involved in viral entry [19]. Sex hormones, including estrogen, can enhance or reduce the expression of cell surface molecules used for viral entry. For example, estrogen alters the expression of the CC chemokine receptors C-C motif chemokine receptor (CCR1 and 5) involved in HIV infection or the integrin αVβ3 that is exploited by adenoviruses for entry [21]. Furthermore, high levels of testosterone have been associated with low neutralizing antibody titers against influenza in men [19, 22]. The differences in the pathogenesis of infectious diseases are therefore known to vary between sexes, but how these sex-dependent differences emerge to modulate vaccine responses is less well understood [23].

## Evidence for sex-based differences in vaccine outcomes

Sex differences in the response to vaccination have been reported both in children and adults [24]. In general, adult females develop higher magnitude immune responses, with respect to antibody levels, and experience more severe adverse events following immunization, due to enhanced immune activation, compared to their male counterparts [13]. Studies in adults and children offer unique opportunities to dissect the impact of sex on vaccine-induced immunity. Specifically, studies on childhood vaccines enable the assessment of sex-dependent response differences without the influence of boosted sex hormones after puberty.

The most common childhood vaccines in the western world are the Bacillus Calmette-Guerin (BCG) vaccine; the combination measles, mumps, and rubella (MMR) vaccine; polio vaccine; and the combination tetanus, diphtheria, and pertussis (DTP) vaccine [25]. The majority of childhood vaccine efficacy studies do not report their findings by sex, so only few, often with conflicting findings, studies on sex differences in infants and children are published [24]. However, sex differences have been noted in the quality of the vaccine-induced immune response to MMR and DTP across the sexes.

DTP side effects differ between sexes, with female infants showing higher rates of hospitalization and mortality after DTP vaccination [26]. In a combination study with DTP, measles and oral polio vaccine (OPV), adverse events were more common in the group receiving all three vaccines, specifically diarrhea and use of medication were increased among girls [27]. The measles-mumps-rubella vaccine, containing three different attenuated virus strains, is administered 12–15 months after birth [28]. Studies pointed to significantly elevated vaccine-specific immunoglobulin G (IgG) titers among girls compared to boys 14 years after vaccination, highlighting longer-term durability and protection among vaccinated girls [29]. Interestingly, another study showed that 2–4 weeks after vaccination (peak immunogenicity), boys had higher antibody responses, but this difference waned 10 weeks post-vaccination [30]. Measles vaccine efficacy against hospitalization was increased in girls as well [31]. In summary, MMR vaccination induces divergent responses in girls and boys, marked by differences in vaccine inflammatory responses and durability. This indicates that differences between the sexes are already evident before puberty, suggesting other influences than pubertal sex hormones on immune tuning.

In adults, as mentioned above, women experience more adverse reactions (AE) following immunization compared to men [32]. One example is following the yellow fever virus (YFV) vaccine, where the subcutaneous application of the live virus is linked to transient viremia and clinical symptoms [33]. The application of this vaccine often leads to strong adverse reactions such as local inflammation, fever, pain, headache, and fatigue [34]. These AEs occur with a higher frequency in women and have been speculated to emerge due to enhanced inflammatory cytokine and chemokine secretion, including tumor necrosis factor (TNF-a), interleukin (IL)-1b, IL-6, and C-X-C motif chemokine 10 (CXCL10) from macrophages and dendritic cells in women [12]. On the other hand, in a second study comparing YFV-vaccinated individuals in the USA and UK, the study documented higher antibody titers in male volunteers and no difference in AEs were observed [35]. Thus, the influence of sex on YFV vaccine-induced AEs and antibody titers remains controversial. However, the differences in AE between males and females did not predict vaccine efficacy, as there was no indication of differences in vaccine-specific humoral responses or protection among sexes [36]. Yet, women have been shown to react more strongly to adjuvants compared to men, potentially related to genetic and hormonal differences in pattern-sensing receptor expression. For example, Toll-like receptor (TLR) agonists as adjuvants are dependent on TLR gene expression, one of which is located on the X chromosome [37]. Specifically, TLR7 ligands, which interact with X-chromosome-encoded TLR7, have been shown to induce higher type 1 IFN production in women, related to differential TLR7 X-chromosome inactivation in women [15]. Thus, given the innate sensing and inflammatory differences among women and men, adjuvant selection may significantly impact future vaccine design, aimed at simultaneously driving robust immunity in the absence of AEs in women.

Seasonal influenza vaccination offers a unique opportunity to study sex-driven differences across ages and seasons. Vaccine efficacy against influenza is measured using a hemagglutination inhibition (HAI) assay, a proxy for viral neutralization, for which titers are elevated in women of all ages [38]. In older women, higher HAI titers have been associated with lower hospitalization and mortality rates compared to men, suggesting that either females maintain higher titers or respond more effectively to vaccination, thereby experiencing better protection in contrast to men [39]. Consistent with other studies, local and systemic reactions after vaccination including muscle pain, redness, and fever were reported to be higher in females [40]. Interestingly, women receiving half the vaccine dose still generate higher immune responses compared to men who received a typical dose [41]. Along the same lines, a mouse study with whole-virus trivalent inactivated influenza vaccine induced higher levels of IgM as well as H1N1-specific IgG1 responses in female mice compared to male mice, which could also be attributable to the enhanced inflammation and AEs observed among females. In conclusion, women may ultimately benefit from reduced vaccine dosing, resulting in reduced AEs, while still inducing high antibody levels.

Interestingly, beyond sex-based vaccine differences, sex also appears to tune disease progression. For example, sex has been profoundly implicated in driving differences in HIV-1 pathogenesis and disease progression [42]. Specifically, even though women have lower plasma viral loads and higher CD4+ T cell counts than men, women have a higher risk of progressing to AIDS (acquired immune deficiency syndrome) [42]. This elevated risk of progression has been linked to elevated immune activation levels in women, thought in part to be driven by enhanced viral RNA sensing in women mediated by TLR7 [43]. Women show higher TLR7-mediated activation of plasmacytoid dendritic cells (pDCs), theorized to account for enhanced inflammation in women [44]. Additional sex-driven differences have been noted in the non-human primate model of HIV. In a simian immunodeficiency virus (SIV) vaccine trial, female macaques were protected more effectively against SIV compared to male animals, and their reduced risk correlated with enhanced mucosal B cell responses [45]. One possible explanation for this difference could be attributable to the elevated IgM and antibody-dependent complement-mediated lytic activity observed in female animals [45]. Moreover, enhanced protection was also associated with differential antibody glycosylation and antibody effector functions across the sexes, pointing to significant differences in the overall quality of the vaccine response across the sexes [45]. Thus, differences between the sexes shape both response to infection and vaccination and track differentially with protection.

Thus collectively, data across vaccines and infections point to significant sex-driven differences in immune programming. The specific immunological cues and mechanisms that selectively result in these immune modulatory effects observed across the genders are beginning to emerge.

## Innate and adaptive immune response differences across the sexes

Differences in vaccine-induced immunity are evident both at the innate and the adaptive immune level. The innate response, our first line of defense against pathogens, is driven by a repertoire of innate immune cells including granulocytes, monocytes, neutrophils, macrophages, dendritic cells (DCs), and natural killer (NK) cells as well as the complement system. These lines of innate immunity act as a non-specific barrier to foreign invaders and are recruited to the site of infection or inflammation by the secretion of cytokines including interleukins, interferons (IFN), and chemokines, where the cells contribute to pathogen destruction or clearance [22]. Conversely, the adaptive immune response develops later, is specific to the pathogen, is long-lived, and provides immunological memory [46]. The adaptive immune response is driven by T and B cells, each contributing to antigen-specific memory through cellular or antibody-mediated control, respectively.

It is known that a sex bias exists in innate immunity at the level of inflammatory cytokine production by antigen-presenting cells like DCs and macrophages [12]. Upon recognition of pathogens or vaccine antigens via pattern recognition receptors like TLRs, pDCs, monocytes, and macrophages produce inflammatory cytokines and chemokines, for example type I interferon to inform the immune system of the presence of a foreign invader [47]. Importantly, elevated type 1 IFN production has been noted in women following vaccination, associated with autoimmune disease [48], and following infection, often linked to TLR7-mediated recognition or related to sex hormone levels [49].

The effects of sex hormones on DCs have been mostly studied in mice, with comparable results in few human studies. Upon exposure to estrogen, immature DCs express elevated levels of IL-6, IL-8, and monocyte chemoattractant protein-1 (MCP-1) as well as enhanced stimulation of T-lymphocytes [50]. Estrogen enhances differentiation of DCs, pro-inflammatory cytokine production, and enhanced expression of major histocompatibility complex (MHCII) [50, 51]. Among DCs, pDCs show the greatest differences following estrogen stimulation between the sexes due to their dominating TLR7 responsiveness [49]. Similarly, macrophages have also been shown to be susceptible to estrogen stimulation [13]. In one study, estrogen was shown to inhibit TNF secretion by monocytes, but this effect was reversed upon stimulation with lipopolysaccharide (LPS) [52]. On the other hand, peripheral monocytes isolated from male subjects produced more TNF-α, IL-1β, and IL-6 [53] but lower amounts of IL-10 compared to cells from females [54]. Beyond cells of the myeloid lineage, NK cells are also influenced by sex hormones. NK cell activity is reduced in menopausal women compared to fertile females [55]. Additionally, contraceptives have been shown to have a significantly inhibitory effect on NK cell function [56]. However, across cell-types, the effect of estrogen on cell activity and cytokine secretion appears to be highly dependent on concentration, where high hormone levels are associated with suppressive activity, while low levels have a limited cytokine stimulatory effect [18]. Conversely, at low hormone levels, females exhibit enhanced antigen presentation capacity and increased phagocytic activity in macrophages and neutrophils [57].

As mentioned above, women mount stronger adaptive humoral and cellular immune responses compared to men [19]. For example, women generate higher antibody responses, marked by higher basal and post-vaccination IgG levels as well as increased B cell numbers in response to vaccination and viral infection [24]. Additionally, non-specific markers of cell-mediated immunity, such as mitogen-stimulated lymphocyte proliferation and wound healing, are upregulated in females [12].

However, beyond differences in vaccine-induced antibody magnitude, recently functional non-neutralizing antibodies have gained importance in vaccinology [2]. Beyond their capacity to bind and neutralize, as mentioned above, antibodies have the capacity to recruit innate immune function, via Fc- and complement receptors present on nearly all immune cells [58]. Specifically, upon binding to target antigens, the antibody Fc-domain can then leverage the anti-pathogen activity of diverse innate immune cells, found within tissue localized compartments to direct pathogen clearance including the recruitment of antibody-dependent cellular cytotoxicity (ADCC), antibody-dependent cellular phagocytosis by neutrophils and monocytes (ADCP), and antibody-dependent complement deposition (ADCD).

Interestingly, significant differences in antibody-mediated functions have been noted across the sexes [59]. Specifically, with respect to antibody response elements, males exhibit enhanced complement activity [60] as well as overall elevated NK cell frequencies [61]. In contrast, females possess more highly phagocytic neutrophils and macrophages [62]. However, differences in antibody activity across the sexes have most carefully been noted following monoclonal therapeutic treatment that requires innate immune system killing to achieve a therapeutic benefit [63]. Specifically, monoclonal antibody (mAb)-mediated immune depletion of B cells has become a central therapeutic approach both in lymphomas and in several autoimmune conditions [64]. Rituximab, a CD20 monoclonal IgG1 antibody, drives B cell depletion in an Fc-receptor-dependent manner [65]. In the oncological space, one study showed that female patients benefit considerably from rituximab treatment compared to standard therapy due to their more active innate responses and their slower therapeutic clearance, and thus longer half-life [66]. Conversely, in the context of rituximab therapy for the treatment of the autoimmune disease, rheumatoid arthritis, men exhibited a better response to rituximab due to reduced inflammation and faster reduction of disease activity compared to women [67]. Thus, sex differences clearly exist in antibody-mediated effector functions across women and men, tuned by sex-dependent differences in response to antibody effector function.

In the setting of vaccination and infection, decreased measles-specific ADCC activity was observed in females, linked to lower sex-specific survival rates [68]. Moreover, elevated ADCC has been linked to slower progression to HIV [69], and HIV-infected cohort studies have shown that HIV-infected men tend to generate higher levels of ADCC than women [69, 70]. Additionally, female mice exhibit lower classical and alternative complement pathway activity [71]. Interestingly, while no sex-dependent differences in concentrations of C1q, mannose-binding lectin (MBL-A), or C3 were found, terminal pathway components, C6 and C9, were reduced in women [71], resulting in differential capacity to activate complement. Moreover, in HIV infection, the ability to generate antibodies able to recruit multiple antibody functions (ADCC, ADCP, and complement) varied between the sexes [45], further highlighting divergence in polyclonal antibody functional activity.

Fc-effector functions can be modulated by subclass selection or via changes in glycosylation of the Fc-domain of the antibody, both of which affect antibody interactions with Fc-receptors [58, 72]. Previous studies have shown that subclass selection varies across the sexes. For example, females infected with human cytomegalovirus (HCMV) elicit higher levels of IgG3, our most functional antibody subclass, compared to men [73]. Female mice immunized with whole-virus trivalent inactivated influenza vaccine (TIV) generate a more robust IgG2a (most functional) response compared to male mice that generate a more balanced IgG2a/IgG1 subclass response [74]. Additionally, female mice also generate a more robust IgM response than males [74]. Along the same lines, IgG subclass profiles in humans against pertussis toxin (PT) and filamentous hemagglutinin (FHA) following pertussis vaccination show higher levels of the poorly functional IgG4 antibody subclass among antigen-specific responses in men [75]. Thus, beyond titer differences, females generate humoral immune responses composed of more inflammatory and functional antibody subclass profiles.

However, in addition to isotype/subclass differences across the sexes, antibody glycosylation also changes dramatically across the sexes [76]. Specifically, galactosylation levels, key to the inflammatory function of antibodies, increase with menopause in women [77], resulting in the generation of less inflammatory antibodies. In contrast, these changes are not observed in men [78]. These data are further supported by the fact that estrogen agonists increase antibody galactosylation (reduced inflammatory agalactosylated antibody levels) [76], highlighting the hormonal dependence of these changes. Moreover, more striking changes in antibody glycosylation occur during pregnancy, at which point the amount of inflamed agalactosylated (G0) antibodies declines during the second and third trimesters of pregnancy. These changes reverse rapidly following birth and have been tightly linked to hormonal changes during the course of pregnancy [79]. Thus collectively, accumulating data point to significant differences in both innate and adaptive immune response across the sexes, including striking differences across the sexes in antibody-mediated functions that may be key to pathogen control and clearance.

## Beyond sex, the impact of pregnancy on humoral immunity

As mentioned above, hormonal changes in pregnancy have been shown to profoundly influence vaccine-induced antibody quality. Critically, immunity shifts to a toleragenic state in the context of pregnancy, critical for the establishment of the fetal graft and maintenance of pregnancy [80]. This shift is manifested as a reduction of inflammatory Th1 responses, resulting in reduced production of IFN-γ and IL-2 [81]. Additionally, within the humoral immune response, pregnancy-related immune changes are accompanied by overall changes in Fc-IgG glycosylation, resulting in increased galactosylation and sialylation, thought to be less inflammatory [77]. Moreover, in addition to the shift in Fc-glycosylation, pregnancy-associated antibody changes are accompanied by a unique increase in the antibody antigen-binding domain (Fab) glycosylation [77]. Specifically, an increased fraction of Fabs are glycosylated during pregnancy, marked by elevated levels of high-mannose structure addition and less mono-sialylated structures [77]. These changes are hypothesized to be mainly caused by elevated levels of progesterone during pregnancy [82] that may lead to unique B cell receptor evolutionary selection. Additionally, changes in glycosylation have also been noted in IgA antibodies, associated with increased IgA bisection during pregnancy whereas IgG bisection is stable, indicating different biological roles of the glycosylated isotypes [83].

Given the observed changes in subclass, isotype, and glycosylation in the humoral immune response, studies have been performed to investigate the overall impact of these changes on disease susceptibility in pregnancy. Studies have demonstrated that pregnant women are more susceptible to infection with influenza virus and are at higher risk for the development of more severe complications such as hospitalization and respiratory illness after infection [84]. This susceptibility has largely been linked to the more attenuated humoral immune profile generated during pregnancy, largely geared towards maintaining the fetal graft. However, the unborn baby relies on the maternal immune response for the transfer of immunity, as maternal antibodies transferred via the placenta represent the primary systemic barrier to infection in early life [80]. While IgGs are the dominant isotype transferred from mother to child, low levels of IgM, IgE, and IgA are transported via the placenta as well. Fetal IgG titers increase over the course of pregnancy, with the most significant increase occurring in the third trimester [85]. Thus, vaccine campaigns aimed at boosting immunity in neonates have empirically focused on vaccination of pregnant mothers, largely in the third trimester of pregnancy [86]. However, given the anti-inflammatory nature of the pregnancy-influenced response, and our limited understanding for the rules by which the placenta sieves antibodies [80], it is uncertain whether traditional vaccine approaches are effectively able to boost the required immunity in neonates. However, next-generation studies aimed at exploring the use of particular adjuvants during pregnancy, aimed at boosting protective antibody titers, linked to our evolving understanding for the mechanisms underlying placental transfer, offer exciting new prospects for the development of custom vaccine approaches to enhance protection of both mothers and their neonates during their time of immune vulnerability.

## Sex-based genetic influences on humoral immune profiles

Beyond the direct influence of sex hormones, gene expression off of the X and Y chromosomes has also been shown to drive immunologic differences in vaccine-induced immunity across the sexes [87]. The X chromosome expresses ten times more genes than the Y chromosome, and many genes on the X chromosome are known to influence immunity [22]. Polymorphisms in Y chromosome genes have been linked to sex-dependent differences in susceptibility to autoimmune diseases like experimental allergic encephalomyelitis, associated with changes in macrophages and NK cell properties [88]. Additionally, epistatic sex-driven differences in HLA allele gene expression have also been linked to higher antibody responses following measles vaccination in girls as compared to boys [88]. Moreover, cytokine gene polymorphisms have also been associated with vaccine response differences across the sexes. Specifically, differences in IL-10 and IL4R gene expression, due to gender-specific polymorphisms, have been associated with changes in antibody responses [13]. Chromosomal mosaicism, caused by random X-chromosome inactivation, is one mechanism by which the female immune response can be shifted due to allele differences. Therefore, the immune response upon X-chromosome silencing in females can vary from males, even within the same family and even across related females [87]. Moreover, many of the genes involved in modulating pathogen- and vaccine-specific immunity are located on the X chromosome or can be modulated in a sex-dependent manner due to estrogen response elements (EREs) in their promotors [19]. These genes include the X-encoded viral sensor TLR7, the T/B cell co-stimulator CD40L, and T regulatory marker FOXP3, all of which play a role in the development of both autoimmunity and pathogen-defense. In X-linked immunodeficiency with hyper-IgM (HIGM1), which is a rare disorder, individuals experience recurring infections associated with very low levels of IgG and IgA and elevated IgM serum titers. This association is explained by a TNF-related activation protein (TRAP), located on the X chromosome, which interacts with its ligand -CD40 on B cells [89]. The resulting failure of TRAP to interact with CD40 on B cells causes the observed immunoglobulin isotype defect in HIGM1 [90], highlighting a unique sex-dependent change in humoral immunity. Therefore, antibody subclass switch and consequently antibody titers are tightly linked to differential X-chromosome expression [91].

Moreover, from a molecular point of view, the X chromosome contains a large number of micro RNAs (miRNAs), known to modulate immunity, while the Y chromosome contains only two. It has been shown that these X-specific miRNAs play a critical role in the development of autoimmune diseases such as lupus, rheumatoid arthritis, and multiple sclerosis–diseases that occur more frequently among women [92]. For example, two specific miRNAs are associated with the induction of the inflammatory cytokine IL-17 as well as upregulation of Tregs and NF-kB signaling pathways in autoimmune diseases [22, 92]. Additionally, CD4+ T cells from female lupus patients express 18 times the normal levels of X-chromosome-linked miRNAs compared to males, indicating an over-activation of T cells in females [48]. Given the known pathogenic role of inflamed antibodies in these autoimmune diseases, it is likely that these inflammatory changes and T cell alterations result in antibody modifications that contribute to disease.

## Microbiome influences on humoral profiles

Finally, another factor that has been linked to altered vaccine and natural-infection humoral profiles is the microbiome [24]. Recent studies have shown that there are sex-specific relationships between the microbiome and the immune response [22]. Moreover, commensal microbial communities can alter sex hormone levels which then regulate autoimmune disease fate and immunological responses to vaccination [93]. Microbes colonize the human gut, skin, oral cavity, and genital area and are well tolerated by the immune system [94]. Diet, age, cultural circumstances, and geographical location all influence our microbiome composition. Specifically, sex hormones impact microbiome composition, as bacteria can metabolize these hormones [95]. Studies on germ-free mice have shown a significant reduction in secretory IgA levels due to the lack of an intestinal microflora, which provides a tonic antigenic stimulus [96]. Likewise, sensing of symbiotic bacteria has been implicated in the induction of IgA class switching as well as localized B cell class switching [97]. Strikingly, response to intra-muscular influenza vaccination in both humans and mice has been linked to immune sampling of microbiome-derived antigens/adjuvants (flagellin) [98], pointing to an intimate interaction between the systemic IgG response to vaccination and the gut microflora. Moreover, probiotics have been shown to boost antibody responses to oral vaccines against salmonella [99] and rotavirus [100]. Given that the microbiome of male and female mice diverge largely after puberty due to hormonal shifts, it is likely that significant differences in the microbiome between the sexes may contribute dominantly to vaccine/pathogen immunity in adult life [12, 95].

## Conclusions and future directions

**Fig. 1:**
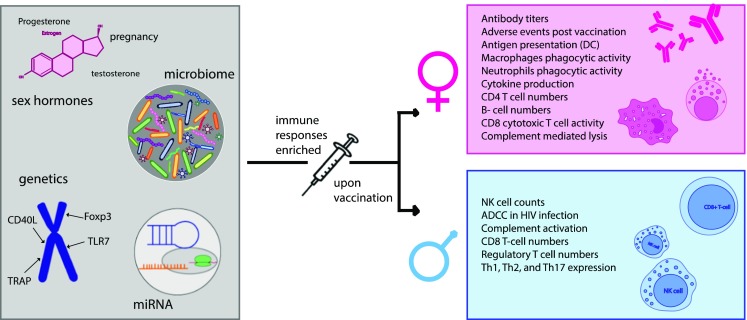
Factors that influence sex-specific humoral immunity to vaccination. Immune response in males and females differ. Females generate higher overall antibody levels, more adverse events, have higher B cell frequencies, exhibit elevated innate immune cell phagocytic activity, etc. (pink box). Males possess increased NK cell numbers, enhanced type-1 immune responses, etc. (blue box). Genetic chromosomal differences, hormone levels, miRNA expression, sex hormones, and gender-specific differences in the microbiome are among some of the factors that underlie differential humoral immunity following vaccination (gray box on the left). However, how these parameters all interact to shape immunity and how they may be harnessed in next generation is incompletely understood

Mounting evidence has shown that sex profoundly influences the immune response and that these sex differences can affect the outcome of vaccinations (Fig. 1). In general, females induce stronger immune functions and higher antibody levels, composed of more functional antibodies, but also experience more adverse reactions to vaccination, linked to a higher probability of developing autoimmune disease. Some of these differences can be linked to hormonal differences, particularly to the level of estrogen, but the lack of age-related differences prior to puberty that exist among the sexes points to additional factors such as miRNAs or other genetic/epigenetic differences between males and females that could influence humoral immunity.

Vaccine efficacy studies often target unique populations determined by geographical location. For example, HIV studies specifically often focus attention on either high-risk group such as (1) men who have sex with men or (2) women in developing countries. While investigators often focus on geographic differences or level of risk, most fail to evaluate responses by sex. Since it has been shown that women have higher immunogenicity and reactogenicity following immunization, and men have higher expression of immune-related proteins, cross-comparisons, a more detailed than simple interrogation of overall magnitude of immunity, might be needed. Refocusing on qualitative differences that have emerged as key mechanistic predictors of protective immunity may pave the way for more effective next-generation sex-specific vaccine design. Yet, despite the growing body of work to support the influence on sex differences on the immune response, most vaccine studies fail to stratify their data by sex. The smaller proportion of women represented in these trials as well as the absence of extensive meta-analysis on sex differences makes it difficult to further investigate differences impacted by sex, as well as the mechanistic underpinnings of these differences. Lack of sufficient statistical power due to limited group size, error rates, and reporting bias of adverse reactions and dietary changes further hamper the research in this field [101]. However, given the differences in AEs and overall immune responses in women, as well as our emerging appreciation for the strong differences in vaccine-induced immune response quality in both pregnant and non-pregnant women, comprehensive dissection of the contributors to these shifts may lead to next generation of rational “sex-specific” vaccine design. These sex-specific vaccine strategies may provide enhanced protection for females in the absence of adverse events and take subgroups such as pregnant women and their unborn children in their first months of life into account while still inducing strong immune reactions in male vaccinees.

Therefore, considering the importance of sex in vaccine response and outcome studies may play a critical part in the design of future vaccine trials. Moreover, understanding the qualitative changes in the humoral immune response among the sexes may provide enhanced resolution of the key vaccine design approaches that may enhance immunity across the hormonal spectrum. Given the profound sex differences across the sexes, it is plausible that unique sex-specific correlates of immunity may even exist. If so, further research may help adapt custom-vaccines for the sexes and improve sex-specific health. Currently, most vaccines are tailored based on a male-dominated participant pool and the same set of vaccines is administered to everyone. Personalized vaccines, customized to address sex-immune profile variation, may offer greater protection against both infectious as well as non-infectious targets [102, 103].
